# The Preventiometer - reliability of a cardiovascular multi-device measurement platform and its measurement agreement with a cohort study

**DOI:** 10.1186/s12874-023-01911-x

**Published:** 2023-04-24

**Authors:** Martin Junge, Markus Krüger, Dietlind L. Wahner-Roedler, Brent A. Bauer, Marcus Dörr, Martin Bahls, Jean-François Chenot, Reiner Biffar, Carsten O. Schmidt

**Affiliations:** 1 Institute for Community Medicine, University of Greifswald, Greifswald, Germany; 2 Present Address: nxt statista GmbH & Co. KG, Hamburg, Germany; 3 Present Address: Unit of Prosthodontics, Gerodontology, and Biomaterials, Centre of Oral Health, University of Greifswald, Greifswald, Germany; 4 Division of General Internal Medicine, Mayo Clinic, Rochester, MN USA; 5 Department of Internal Medicine B (Cardiology), University Medicine, Greifswald, Germany; 6 German Centre for Cardiovascular Research (DZHK), Partner Site Greifswald, University of Greifswald, Greifswald, Germany; 7 Department of General Practice, Institute for Community Medicine, University of Greifswald, Greifswald, Germany; 8 Unit of Prosthodontics, Gerodontology, and Biomaterials, Centre of Oral Health, University of Greifswald, Greifswald, Germany

**Keywords:** Method-comparison studies, Agreement, Reliability, Validity, Measurement, Bland-Altman Plots

## Abstract

**Background:**

Multimedia multi-device measurement platforms may make the assessment of prevention-related medical variables with a focus on cardiovascular outcomes more attractive and time-efficient. The aim of the studies was to evaluate the reliability (Study 1) and the measurement agreement with a cohort study (Study 2) of selected measures of such a device, the Preventiometer.

**Methods:**

In Study 1 (*N* = 75), we conducted repeated measurements in two Preventiometers for four examinations (blood pressure measurement, pulse oximetry, body fat measurement, and spirometry) to analyze their agreement and derive (retest-)reliability estimates. In Study 2 (*N* = 150), we compared somatometry, blood pressure, pulse oximetry, body fat, and spirometry measurements in the Preventiometer with corresponding measurements used in the population-based Study of Health in Pomerania (SHIP) to evaluate measurement agreement.

**Results:**

Intraclass correlations coefficients (ICCs) ranged from .84 to .99 for all examinations in Study 1. Whereas bias was not an issue for most examinations in Study 2, limits of agreement for most examinations were very large compared to results of similar method comparison studies.

**Conclusion:**

We observed a high retest-reliability of the assessed clinical examinations in the Preventiometer. Some disagreements between Preventiometer and SHIP examinations can be attributed to procedural differences in the examinations. Methodological and technical improvements are recommended before using the Preventiometer in population-based research.

**Supplementary Information:**

The online version contains supplementary material available at 10.1186/s12874-023-01911-x.

## Introduction

Over the last 50 years, the number and complexity of epidemiologic studies has grown and demands for participants has risen [1]. However, willingness to volunteer for scientific activities has declined, which is reflected by decreasing response rates [1–3]. Therefore, an initial refusal to participate in a study may not be interpreted as a general refusal of taking part in the study itself. Rather, constraints on participants’ time and availability might make study demands appear too high. Therefore, making clinical examinations more efficient and attractive, using multimedia options, and making such offers closer to the participants’ place of residence in a digital form or mobile platform might improve participation rates.

**Fig. 1:**
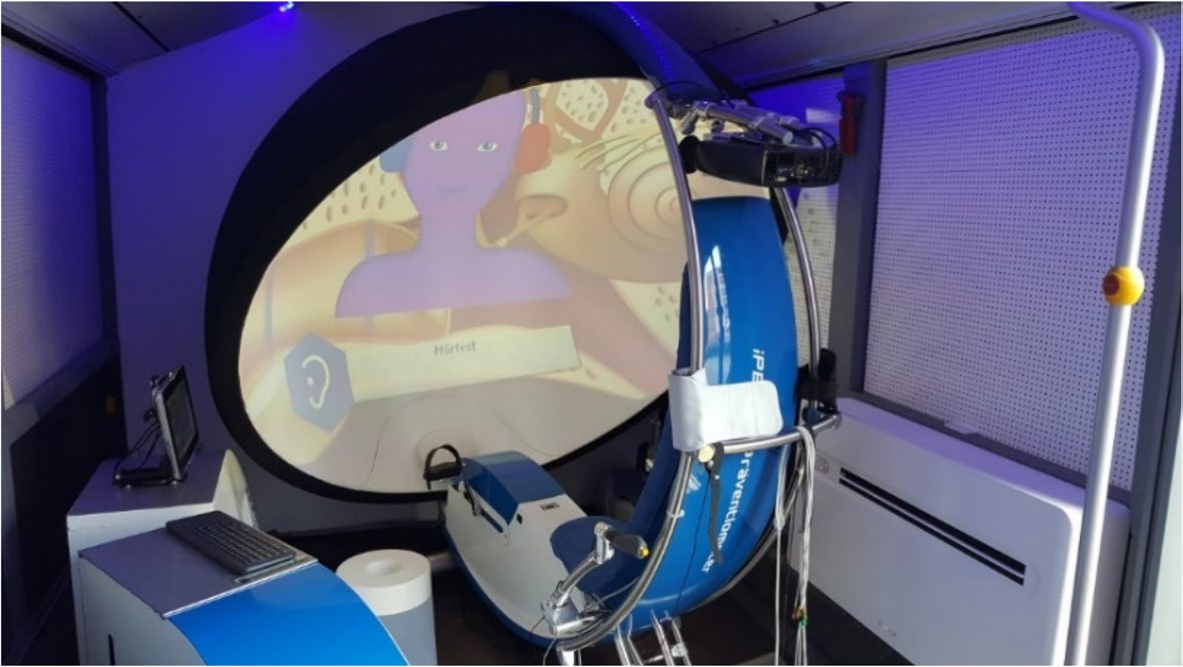
The mobile Preventiometer installed in a bus (Preventiometer 1)

Digital solutions are already in use for survey-based research and are also increasingly applied to patient-reported outcome measures (PROMs). Beyond self-reported measures, wearables and smartphone applications are promising candidates that may also facilitate mobile measurement of medical variables [4, 5]. Another approach is taken by the *Preventiometer* (Fig. 1) [5, 6]. It is an interactive multi-device platform designed to assess prevention-related medical variables such as blood pressure, body fat, and pulse oximetry. During examinations, the participant takes place in a padded seat and looks at the inner side of a dome where videos are projected to (see Fig. 1). These videos contain instructions and background information on the examinations. The procedure can be controlled by the participant by pressing two buttons integrated into the armrest of the seat. The entire examination is accompanied by a study nurse who operates the control computer of the Preventiometer and monitors the measurement processes. The Preventiometer can be implemented in a mobile platform (e.g. a bus or van) to enable examinations closer to the participants place of residence. While the virtual assistant may contribute to a higher degree of standardization, the uncommon examination environment might also induce excitement, thereby impacting clinical measurements.

Acceptance of the Preventiometer by participants was previously assessed in a wellness context at Mayo clinics [7, 8]. Participants agreed or strongly agreed that it was both comfortable and engaging. In our current project ***P*** *rävention für* ***A*** *rbeitnehmer zur Reduktion von* ***K*** *rankheits* ***t*** *agen durch* ***M*** *otivation und* ***V*** *erhaltensänderung* ([preventive healthcare for workers with the aim to reduce absenteeism by motivation and behavior] PAKt-MV) [9] we evaluated the accuracy of the central measurement device as related results were not available from other studies.

In Study 1, we estimated the reliability by measuring participants twice within a Preventiometer and assessed the agreement between the repeated measurements. In Study 2, we estimated the measurement agreement of the Preventiometer with results of similar variables as obtained in the examination center of a population based cohort study, the Study of Health in Pomerania (SHIP) [10–12]. In both studies, only those examinations and variables of the Preventiometer that had a comparable examination in SHIP were included.

## Study 1: agreement of repeated measurements within Preventiometers (Reliability)

The goal of Study 1 was to estimate the reliability of Preventiometer. Two Preventiometers at different locations in different environments were used in this study. One was on a mobile platform placed in a bus and the other one was stationary in a room of the local hospital. A stationary Preventiometer was used because only one bus was available. Participants were tested twice (repeated measure) with one of the Preventiometers. For efficiency and comparability between Study 1 and Study 2 we selected only measures that were available for the Preventiometer and SHIP for Study 1.

### Materials and methods

#### Study sample

A convenience sample of 22 males and 53 females with a mean age of 41.7 years (*SD* = 13.3) in the range from 18 to 71 years participated. All participants were recruited among employees of the University Medicine of Greifswald and their families or acquaintances. All participants gave written informed consent. The Ethics Committee of the University Medicine Greifswald approved the study protocol.

#### Preventiometer

Two Preventiometers were used for the reliability assessment. The first Preventiometer was installed in an articulated bus (Mercedes-Benz Citaro G, Evobus) at the premises of the University Medicine Greifswald as part of the mobile preventive healthcare project PAKt-MV. It will be referred to as the *mobile* Preventiometer. The second Preventiometer was installed in an office within the Department of General Practice. It will be referred to as the *stationary* Preventiometer. Five examinations of the Preventiometer were comparable to examinations of SHIP (see Study 2): Somatometry, blood pressure measurement, body fat measurement, pulse oximetry and spirometry (see Table 1 for a detailed overview). Because somatometric examinations were conducted outside the Preventiometer device, they were only assessed once and are therefore not subject to reliability analysis.

**Table 1:** Comparable examinations and the corresponding measurement instruments of the Preventiometer and SHIP

Examination	Variable	Preventiometer	SHIP
*Somatometry*	Body height	Seca 213 stadiometer	Body length measuring device SOEHNLE
	Body weight	A&D UC-321PL	SOEHNLE S20
	Waist circumference	Seca 201 tape	Schneider tape
	Hip circumference		
*Blood pressure measurement*	Systolic blood pressure	HealthGuard-15 Portable Health Kiosk OEM	Omron 705 IT
	Diastolic blood pressure		
*Body fat measurement*	Body fat (%)	Futrex PM 860	Bod Pod (Cosmed)
*Pulse measurement*	Heart rate	Nonin 3231 USB	Omron 705 IT
*Spirometry*	Peak Flow (PEF)	Carefusion SpiroUSB	Viasys Healthcare Masterscreen
	Vital capacity (FVC)		

Examinations within the Preventiometer were conducted by study nurses who were first trained in the SHIP examination center for basic examinations (somatometry, blood pressure measurement, and spirometry) and then trained by instructors from the manufacturer of the Preventiometer.

#### Design

Study 1 followed a repeated measurement design, i.e. each participant was examined twice in a Preventiometer in immediate succession. The examinations within Preventiometers were always conducted in the following order: Somatometry (only at the first measurement occasion), blood pressure and body fat measurement, pulse oximetry and spirometry. A subset of the participants (*n* = 22 with a mean age of 32.7 [SD = 8.65], consisting of 7 males and 15 females) were examined *twice* in *each* Preventiometer in immediate succession, thus contributing data for the analysis of both Preventiometers (in contrast to participants that were tested *twice* in *one* of the Preventiometers). The clinical measurements in the Preventiometer are described in detail below.

##### *Somatometry*

Height was measured using a stadiometer. Participants were asked to remove their shoes for this measurement. For the waist and hip circumferences a simple measuring tape was used. For the weighting participants stripped down to their underwear.

##### Blood pressure and body fat measurement

Systolic and diastolic blood pressure and body fat percentage were both measured with the OEM version of the HealthGuard-15 Portable Health Kiosk. It consists of an oscillometric blood pressure measurement device and a near-infrared interactance body fat measurement device [13]. The cuff for the blood pressure measurement was applied to the left and the body fat sensor to the triceps of the right arm of the participant. Both measurements were taken simultaneously. This measurement was taken after non-exhausting activities (i.e., somatometry), but no specified resting phase was implemented. This procedure followed the suggestions by the manufacturer.

##### *Pulse oximetry*

For pulse oximetry, a Nonin 3231 USB Pulse oximeter was used that was attached to the right index finger of the participant.

##### *Spirometry*

Spirometric parameters were measured with the Carefusion SpiroUSB spirometer. At least three expiratory maneuvers were conducted from which the best trial was selected to determine the spirometric parameters of interest. The procedure followed a detailed SOP that was in line with German guidelines [14] as far as the expiratory part of spirometry is concerned.

#### Statistical analysis

We evaluated the reliability of measurements by means of intra-class correlation coefficients (ICC) as a two-way random effects model with absolute agreement and single measurement [15]. We considered ICCs ≥ 0.70 as indicative of acceptable reliability [16]. Additionally, we report the variance components (VC) for persons, replications and residuals estimated by the ICC function from the R package *psych* to allow for a differentiation of systematic and random measurement error and the standard error of measurement for agreement (SEM_agreement_) as proposed by Vet et al. [17]. Furthermore, we computed the mean of differences (i.e. bias) between repeated measurements within participants, the standardized mean difference (SMD), and the limits of agreement (LoA) for the repeated measurements. The SMD was computed as the mean of the differences (i.e. bias) between repeated measurements within participants divided by the standard deviation of these differences, and the limits of agreement were computed as the mean of the differences (i.e. bias) ± 1.96 times the standard deviation of the differences between the first and second measurements.

Finally, we plotted the differences against the averages according to Bland and Altman [18] to allow for a visual inspection of (dis-)agreement between the measurements. All analyses were conducted separately for the mobile and the stationary Preventiometer.

All data were complete. All calculations were performed with the statistical software R [19] and additional R packages [20–25].

### Results

**Fig. 2:**
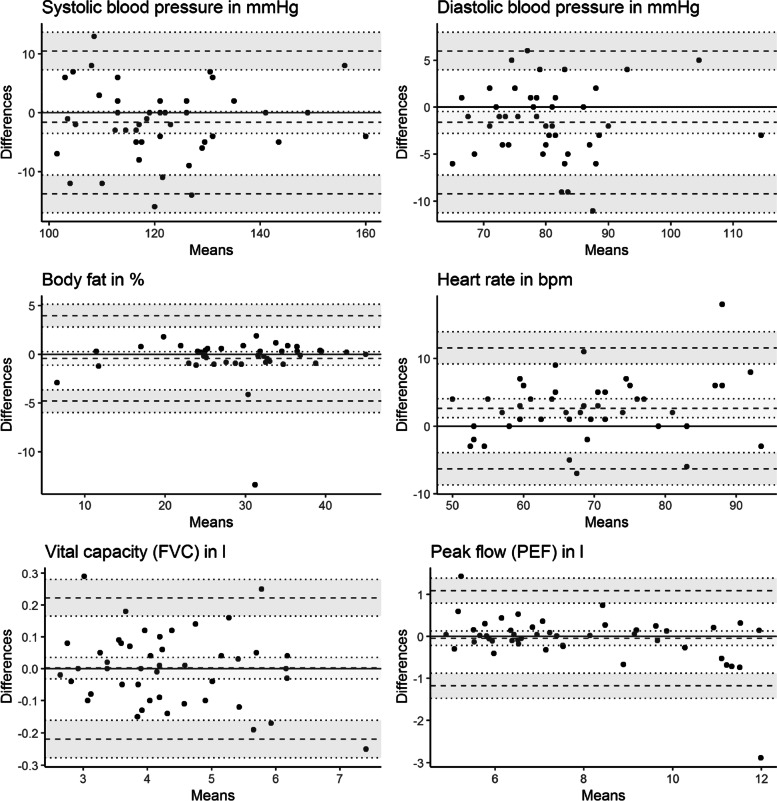
Bland–Altman Plots for repeated measurements within Preventiometer 1 (mobile)

**Fig. 3:**
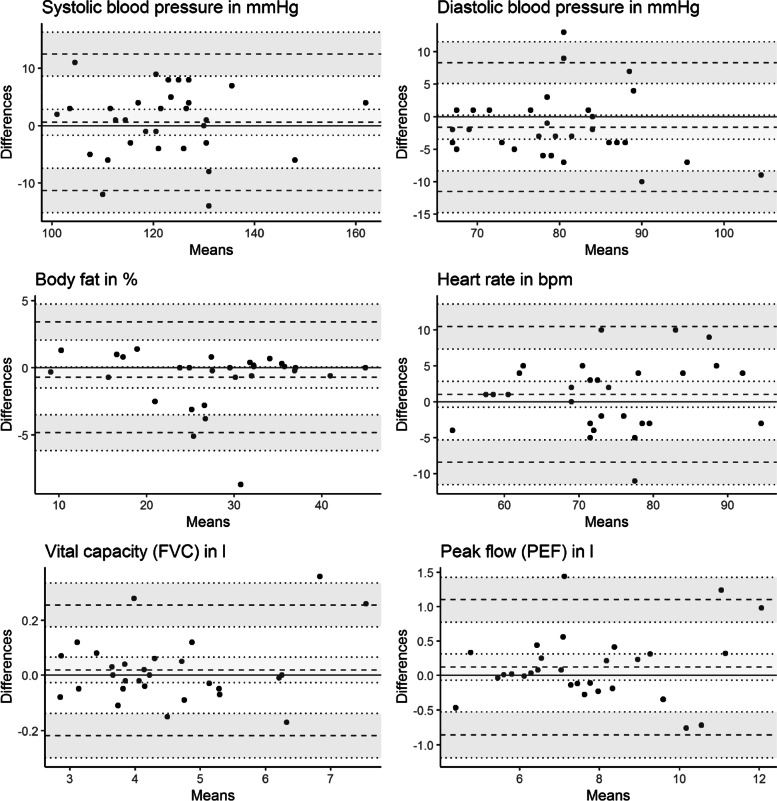
Bland–Altman Plots for repeated measurements within Preventiometer 2 (stationary)

All examinations have ICCs above 0.70 (see Table 2). ICCs for diastolic blood pressure (mobile), body fat, heart rate (mobile) and spirometric variables surpass 0.90. There are no substantial *mean differences* between the first and second measurement in the Bland–Altman-plots (see Fig. 2 and Fig. 3). However, observed extreme differences between observations primarily concerned the mobile Preventiometer. This is also in line with the tendency of the variance component of the replications to be higher for the mobile Preventiometer in the case of blood pressure and heart rate measurements.

**Table 2:** Agreement between repeated measurements for mobile and stationary Preventiometers

Variable	n	1^st^ Meas M (SD)	2^nd^ Meas M (SD)	Mean difference [CI]	SMD	Lower LoA [CI]	Upper LoA [CI]	ICC [CI]	VC persons	VC methods	VC residuals	SEM agreement
Systolic blood pressure in mmHg												
*Mobile*	45	120.5 (13.8)	122.1 (13.8)	1.6 [-0.2; 3.5]	0.27	-13.8 [-17; -10.6]	10.5 [7.3; 13.7]	.90 [.82; .94]	171.96	0.93	19.21	4.49
*Stationary*	31	122.5 (12.9)	121.9 (13.2)	-0.6 [-2.8; 1.6]	-0.1	-11.3 [-15.1; -7.5]	12.5 [8.6; 16.3]	.89 [.79; .95]	152.14	0	17.94	4.24
Diastolic blood pressure in mmHg												
*Mobile*	45	79.5 (9.4)	81.1 (9.6)	1.6 [0.5; 2.8]	0.42	-9.2 [-11.2; -7.2]	6 [4; 8]	.91 [.82; .95]	82.68	1.15	7.48	2.94
*Stationary*	31	79.1 (8.8)	80.7 (9.6)	-1.6 [-0.2; 3.5]	0.32	-11.5 [-14.7; -8.3]	8.3 [5.1; 11.5]	.84 [.69; .92]	71.96	0.89	12.82	3.70
Body fat in %												
*Mobile*	45	29.6 (8.7)	30 (8.6)	0.4 [-0.3; 1.1]	0.19	-4.8 [-6; -3.6]	4 [2.8; 5.1]	.97 [.94; .98]	71.78	0.03	2.51	1.59
*Stationary*	31	28.3 (9.5)	29.1 (9.5)	0.7 [-0.1; 1.5]	0.34	-4.8 [-6.2; -3.5]	3.4 [2.1; 4.7]	.97 [.94; .99]	87.69	0.19	2.21	1.55
Heart rate in bpm												
*Mobile*	44	70.2 (11.9)	67.6 (10.9)	-2.6 [-4; -1.2]	-0.58	-6.3 [-8.7; -3.9]	11.6 [9.2; 14]	.90 [.75; .95]	120.33	3.24	10.40	3.69
*Stationary*	30	74.2 (10.6)	73.1 (10)	-1 [-2.8; 0.8]	-0.21	-8.4 [-11.5; -5.3]	10.5 [7.4; 13.6]	.89 [.78; .95]	94.77	0.15	11.60	3.43
Vital capacity (FVC) in l												
*Mobile*	46	4.3 (1.1)	4.3 (1.1)	0 [0; 0]	-0.01	-0.2 [-0.3; -0.2]	0.2 [0.2; 0.3]	.99 [.99; 1]	1.20	0	0.01	0.08
*Stationary*	29	4.5 (1.2)	4.5 (1.2)	0 [-0.1; 0]	-0.16	-0.2 [-0.3; -0.1]	0.3 [0.2; 0.3]	1 [.99; 1]	1.44	0	0.01	0.08
Peak flow (PEF) in l												
*Mobile*	46	7.8 (2.1)	7.8 (2.3)	0 [-0.1; 0.2]	0.08	-1.2 [-1.5; -0.9]	1.1 [0.8; 1.4]	.97 [.94; .98]	4.63	0	0.17	0.41
*Stationary*	29	7.8 (2)	7.7 (1.9)	-0.1 [-0.3; 0.1]	-0.24	-0.9 [-1.2; -0.5]	1.1 [0.8; 1.4]	.97 [.93; .98]	3.79	0	0.12	0.36

*Meas.* Measurement, *M* mean, *SD* standard deviation, *CI* confidence interval, *LoA* limit of agreement, *ICC* intraclass correlation coefficient, *VC* variance components, *SEM* standard error of measurement

### Discussion

In both Preventiometers, retest-reliability estimates were excellent for *body fat*, *vital capacity*, and *peak flow* whereas agreement for the *systolic blood pressure*, *diastolic blood pressure* and *heart rate* was lower but still in the acceptable range [16].

To put our result in context, we compared them with results from other reliability studies (Table 3). Overall, reliability in terms of ICCs are mostly in line with comparable method comparison studies and can be regarded as sufficient, yet some discrepancies are noteworthy. For example, in the context of the HERITAGE family study [26], ICCs for blood pressure were somewhat smaller than in our study. This may be explained by the larger time interval between measurements in the HERITAGE study (one day vs. approximately one hour). The ICCs from a study evaluating the reliability of a predecessor of the body fat measurement device built into the Preventiometer [27] were slightly smaller than in our study. ICCs for heart rate measurements in our study lie in the middle of the range of ICCs that have been reported in two studies comparing different devices for the measurement of *heart rate* [4, 28]. Whereas the ICCs for Peak flow (PEF) are in line with observed ICCs from other studies [29, 30], ICCs for FVC in our study are larger. This may be due to the shorter time interval between both measurements. Overall, the mean differences between the first and second measurements were small. Foremost in the mobile Preventiometer, heart rate seems to decrease slightly between the first and second measurement. This may reflect an adaptation to the new and mildly exciting examination context in the mobile Preventiometer.

**Table 3:** Reliability estimates from similar method comparison studies

Study	Devices	Comparison	Variable	n	ICC
Burkard et al. 2018 [31]	Omron HBP-1300	First vs. mean of second to fourth measurement	Systolic BP	802	.85
			Diastolic BP	802	.87
Stanforth et al. 2000 [26]	Colin STBP-780	Means of measurements (4 – 8) between two consecutive days	Systolic BP	822	.84
			Diastolic BP	822	.79
Nielsen et al. 1992 [27]	Futrex-5000	within-day	% body fat	34	.91
		between-day	% body fat	34	.95
Mitchell et al. 2016 [4]	Android app	Test–retest with 5 min interval	Heart rate	111	.82
	iOS app		Heart rate	111	.76
	Polar watch		Heart rate	111	.84
Losa-Iglesias et al. 2016 [28]	Radial pulse	Test–retest with 3 consecutive measurements	Heart rate	46	.99
	Pulsoximeter		Heart rate	46	.99
	App		Heart rate	46	.97
Krug et al. 2011 [29]	not specified	Repeated measurement between 3 to 16 days apart	FVC	633	.87
Fonseca et al. 2005 [30]	PiKo-1	Within-session reliability of two best maneuvers	PEF	38	.96
	Spirotel		PEF	38	.97
	Mini-Wright		PEF	38	.95

## Study 2: agreement between Preventiometer and SHIP measurements (validity)

The aim of Study 2 was to estimate the measurement agreement of Preventiometer examinations with comparable examinations in a population-based cohort study, the Study of Health in Pomerania (SHIP). This provides insights into the usability of Preventiometer measurements instead of SHIP measurements, for example when potential participants can better be accessed by allowing for a mobile assessment close to their homes. SHIP comprises two cohorts, and a large range of health related variables have been assessed. More details have been described elsewhere [10–12]. SHIP is subject to rigorous internal and external quality control Therefore, data from SHIP was used as reference for the Preventiometer.

### Materials and methods

#### Study sample

In total, 155 (53% female) participants of the SHIP-Trend-1 cohort [11] with a mean age of 57 years (*SD* = 13) were enrolled. Recruitment for additional Preventiometer assessments took place at the SHIP examination center after participants completed their SHIP examinations on the same day.

All participants gave written informed consent. The Ethics Committee of the University Medicine Greifswald approved the study protocol.

#### Design

The design of Study 2 followed a method comparison study design with a single measurement on each method [32]. Participants were first examined in the SHIP study center and afterwards in one of the two Preventiometers. The time interval between the two measurements was about 1 to 6 h. Examinations in SHIP were conducted by certified SHIP examiners whereas examinations in the Preventiometers were performed by examiners of the project PAKt-MV who were trained both in the SHIP study center and on the Preventiometer.

#### Examinations

Examinations of the Preventiometer have been described in the methods section of Study 1. Detailed descriptions of SHIP examinations can be found elsewhere (e.g., blood pressure, height, weight, and waist circumference [33]; spirometry [34, 35]). A comparison of the instruments is displayed in Table 1. In the following section, we focus on methodological differences between Preventiometer and SHIP that might be of relevance for the evaluation of their agreement.

##### *Somatometry*

Whereas body height is measured with a mechanical stadiometer in the Preventiometer, it is measured via an ultrasound method in SHIP. Weight and waist circumference variables are measured using similar measurement techniques (see Table 1). Participants were asked to take off their shoes for height measurement and strip to their underwear for weight measurement.

##### *Blood pressure measurement*

Blood pressure is measured in the Preventiometer and SHIP by automatic oscillometric devices. However, in the Preventiometer, blood pressure is measured once without an explicit resting phase before the measurement, while blood pressure is measured three times in SHIP and the final value is computed as the mean of the second and third measurement. Before the first measurement, there is a five-minute resting phase in SHIP and between the three measurements, there are three minutes pauses. Finally, in the Preventiometer, blood pressure is measured on the left arm whereas in the SHIP, blood pressure is measured on the right arm.

##### *Body fat measurement*

Body fat percentage is measured by a near infrared interactance device in the Preventiometer where a sensor is placed on the triceps of the participant. On the basis of this measurement, the fat percentage of the whole body is extrapolated. In contrast, in SHIP body fat percentage is measured using a Bod Pod, which uses air displacement plethysmography [36–39].

##### *Pulse oximetry*

Heart rate is measured by a pulse oximeter in the Preventiometer. In SHIP, heart rate is determined during the course of blood pressure measurement by the blood pressure device.

##### *Spirometry*

The spirometry device in the Preventiometer only recorded expiratory maneuvers but did not allow measurements of inspiratory maneuvers while in SHIP, an inspiratory and an expiratory maneuver was conducted.

#### Statistical analysis

We evaluated the agreement between measurements analogous to Study 1. Again, all analyses were conducted separately for the mobile and the stationary Preventiometer.

We excluded five data pairs from the analyses. In two cases, body weight was measured fully clothed in the Preventiometer which violated the study protocol. In another two cases, extreme differences for body height measurement (128.2 cm in the Preventiometer vs. 168 cm in the SHIP and 159.5 cm in the Preventiometer vs 170 cm in the SHIP, respectively) were most likely due to data input errors in the Preventiometer. Finally, an extremely large difference for body weight measurement was detected (81.9 kg in the Preventiometer vs. 112.9 kg in the SHIP). This was also attributed to a data input error in the Preventiometer. Additionally, there were a few missing comparisons per examination (see Table 4) which were due to occasional malfunctions of the Preventiometer and missing values in the SHIP. All calculations were performed with the statistical software R [19] and additional packages [20–25].

**Table 4:** Agreement between Preventiometer (mobile and stationary) and SHIP measurements

Variable	n	Prev M (SD)	SHIP M (SD)	Bias [CI]	SMD	Lower LoA [CI]	Upper LoA [CI]	ICC [CI]	VC persons	VC methods	VC residuals	SEM agreement
Body height in cm												
*Mobile*	44	171 (9.2)	170.8 (8.8)	-0.2 [-0.5; 0]	-0.25	-1.4 [-1.8; -1]	1.8 [1.4; 2.3]	1 [.99; 1]	80.83	0.01	0.34	0.59
*Stationary*	102	171.2 (9.4)	171.1 (9.1)	-0.1 [-0.3; 0.2]	-0.06	-2.1 [-2.5; -1.8]	2.3 [1.9; 2.7]	.99 [.99; .99]	85.29	0	0.64	0.80
Body weight in kg												
*Mobile*	44	83.7 (16.1)	82.6 (16)	-1.1 [-1.4; -0.8]	-1.13	-0.8 [-1.4; -0.3]	3.1 [2.6; 3.6]	1 [.94; 1]	257.04	0.63	0.50	1.06
*Stationary*	101	85.6 (16)	85.2 (16.1)	-0.4 [-0.5; -0.2]	-0.56	-1 [-1.2; -0.7]	1.7 [1.5; 1.9]	1 [1; 1]	257.21	0.07	0.23	0.55
Waist circumference in cm												
*Mobile*	46	94.5 (13.9)	96.6 (13.2)	2.1 [0.9; 3.4]	0.5	-10.4 [-12.6; -8.3]	6.2 [4; 8.4]	.94 [.87; .97]	175.65	2.05	9	3.32
*Stationary*	100	97.7 (14.9)	99.1 (14.6)	1.4 [0.5; 2.3]	0.3	-10.5 [-12.1; -8.9]	7.7 [6.1; 9.2]	.95 [.92; .97]	207.55	0.89	10.73	3.41
Hip circumference in cm												
*Mobile*	46	101.4 (10.4)	101.8 (10.5)	0.4 [-1; 1.7]	0.08	-9.2 [-11.5; -6.9]	8.5 [6.2; 10.8]	.91 [.84; .95]	99.17	0	9.97	3.16
*Stationary*	100	103.3 (10.8)	103.7 (10.7)	0.4 [-0.3; 1.2]	0.11	-7.9 [-9.2; -6.6]	7 [5.7; 8.3]	.94 [.91; .96]	107.58	0.02	7.26	2.70
Systolic blood pressure in mmHg												
*Mobile*	44	123.6 (14.7)	121.6 (13.1)	-2.1 [-4.8; 0.6]	-0.23	-15.4 [-20.1; -10.7]	19.6 [14.9; 24.2]	.79 [.65; .88]	153.44	1.23	39.79	6.41
*Stationary*	99	131.9 (17.7)	127.8 (16.9)	-4.2 [-6.8; -1.5]	-0.31	-21.9 [-26.5; -17.4]	30.3 [25.7; 34.8]	.69 [.55; .78]	211.07	7.78	88.68	9.82
Diastolic blood pressure in mmHg												
*Mobile*	44	80.2 (11.1)	72.8 (8.5)	-7.4 [-9.9; -4.8]	-0.88	-9.1 [-13.5; -4.7]	23.9 [19.5; 28.3]	.50 [.04; .75]	62.75	26.47	35.36	7.86
*Stationary*	99	80.3 (8.9)	75.2 (9.6)	-5.2 [-6.6; -3.7]	-0.7	-9.3 [-11.8; -6.8]	19.6 [17.1; 22.1]	.59 [.25; .77]	58.09	13.02	27.19	6.34
Body fat in %												
*Mobile*	33	33.3 (7.8)	33.3 (9.4)	0 [-1.7; 1.7]	0	-9.5 [-12.4; -6.5]	9.5 [6.5; 12.4]	.85 [.71; .92]	62.59	0	11.31	3.36
*Stationary*	97	33.2 (8.6)	34.9 (10)	1.7 [0.6; 2.8]	0.3	-12.7 [-14.6; -10.8]	9.3 [7.4; 11.2]	.81 [.71; .87]	70.81	1.29	15.73	4.13
Heart rate in bpm												
*Mobile*	44	74.9 (11.4)	72.3 (11.6)	-2.6 [-4.9; -0.2]	-0.33	-12.6 [-16.7; -8.6]	17.8 [13.7; 21.8]	.76 [.59; .86]	101.75	2.61	30.10	5.72
*Stationary*	99	70 (10.9)	70.1 (9.4)	0.2 [-1.4; 1.7]	0.02	-15.3 [-18; -12.7]	15 [12.4; 17.6]	.71 [.60; .80]	73.09	0	29.63	5.44
Vital capacity (FVC) in l												
*Mobile*	37	4 (0.9)	4.2 (1)	0.2 [0.1; 0.3]	0.58	-0.9 [-1.1; -0.7]	0.5 [0.3; 0.7]	.92 [.80; .97]	0.90	0.02	0.06	0.27
*Stationary*	86	4 (1.1)	4.1 (1.2)	0.1 [0.1; 0.2]	0.51	-0.7 [-0.8; -0.6]	0.4 [0.3; 0.5]	.97 [.93; .98]	1.23	0.01	0.04	0.21
Peak flow (PEF) in l												
*Mobile*	37	7 (1.9)	7.2 (2.2)	0.2 [-0.2; 0.6]	0.17	-2.4 [-3.1; -1.8]	2 [1.4; 2.7]	.85 [.72; .92]	3.54	0	0.64	0.80
*Stationary*	86	7.4 (2.3)	7.5 (2.3)	0.1 [-0.2; 0.4]	0.09	-2.6 [-3; -2.1]	2.3 [1.9; 2.8]	.85 [.78; .90]	4.57	0	0.79	0.89

*n* number of participants, *Prev.* Preventiometer, *M* mean, *SD* standard deviation, *CI* confidence interval, *SMD* standardized mean difference, *LoA* limit of agreement, *ICC* intraclass correlation coefficient, *VC* variance components, *SEM* standard error of measurement

### Results

All ICCs were larger than 0.70, except for *systolic blood pressure* in the stationary Preventiometer and *diastolic blood pressure* in both Preventiometers.

Positive bias (i.e., Preventiometer measurements larger than SHIP measurements on average) were found for *body height*, *body weight*, *systolic* and *diastolic blood pressure* and *heart rate* (mobile Preventiometer). Negative bias (i.e., Preventiometer measurements smaller than their SHIP counterparts on average) were found for *waist* and *hip circumference*, *vital capacity* and *peak flow* and *heart rate* (stationary Preventiometer).

**Fig. 4:**
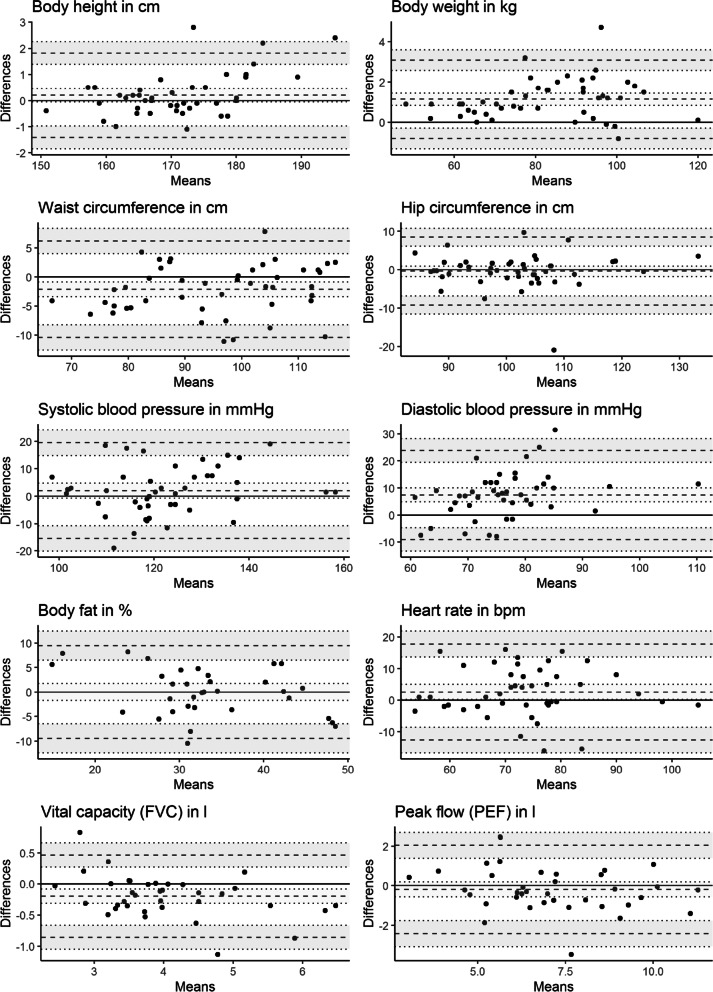
Bland–Altman plots for the comparison between Preventiometer 1 (mobile) and SHIP measurements

**Fig. 5:**
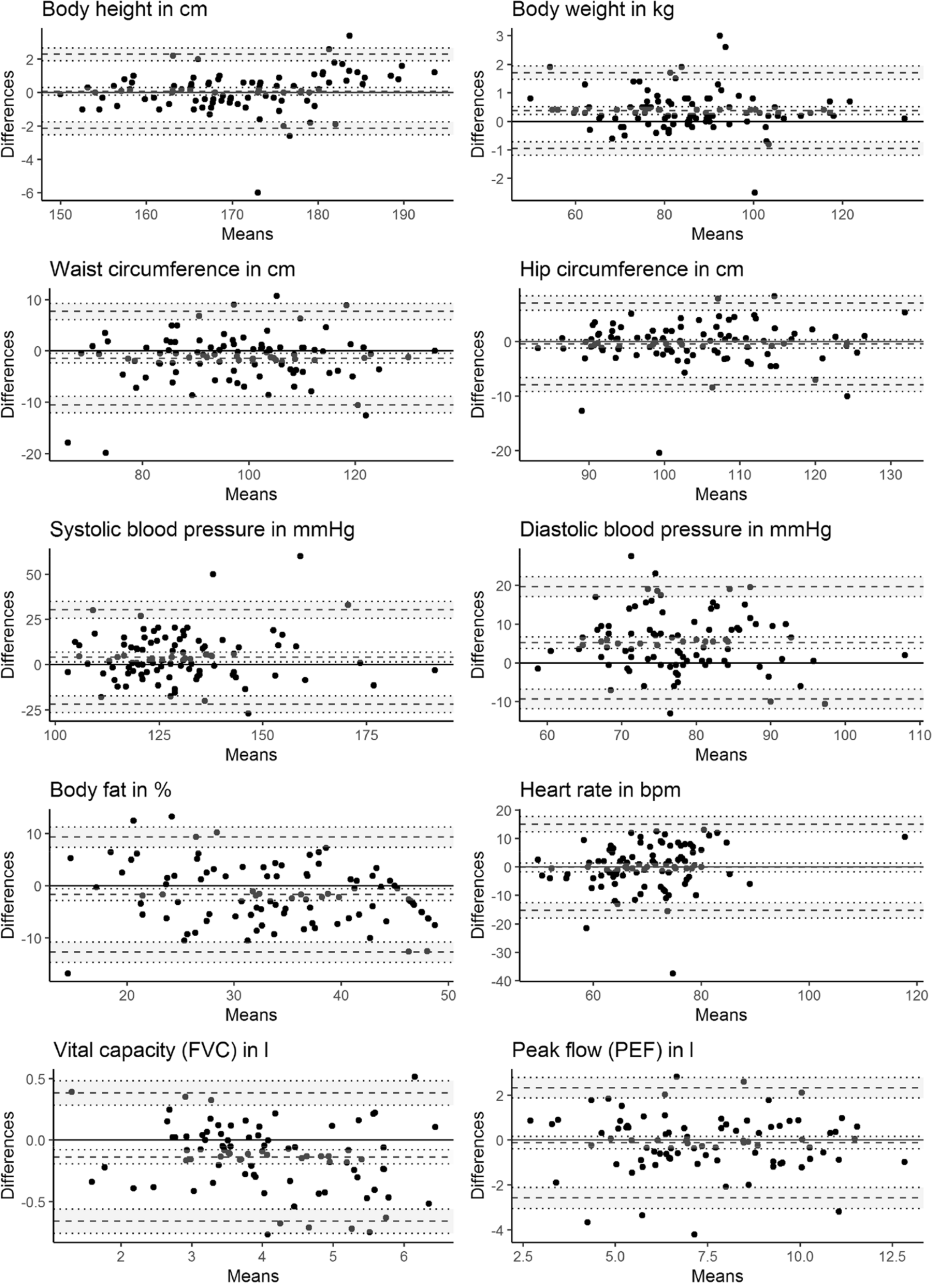
Bland–Altman plots for the comparison between Preventiometer 2 (stationary) and SHIP measurements

Comparing the Bland–Altman Plots for *hip* and *waist circumference* for the mobile Preventiometer (Fig. 4), the size of the LoA for hip circumference measurements is mainly driven by some extremely large differences, even after the outlier elimination, whereas the range of the LoA for *waist circumference* measurements is based on a more consistent distribution of the differences. There is also evidence for proportional bias (i.e. a statistically significant slope in the regression of the differences on the averages) in the Bland–Altman plots of *body height*, *diastolic blood pressure*, *body fat* and *vital capacity* for the mobile Preventiometer. Regarding the stationary Preventiometer (Fig. 5), some extreme differences between measurements occurred that are located by a far margin outside the limits of agreement. In the cases of *hip* and *waist circumference* measurements, differences around 20 cm occurred. For *systolic blood pressure* measurement, there are two differences around or even above 50 mmHg. This is also reflected in a much higher variance component of methods for the stationary Preventiometer for these measurements. Furthermore, there is evidence for proportional bias (see above) for *body height*, *heart rate*, *body fat* and *vital capacity*.

### Discussion

In Study 2, we assessed measurement agreement from a mobile and a stationary Preventiometer with measurements obtained during SHIP examinations. While SHIP measurements can be conceived as a proxy to validity, there are two concerns that limit this interpretation: (1) Some of the measures change over the course of the day, such as blood pressure. There were up to several hours between both measurements because participants were first fully examined in SHIP and afterwards in one of the Preventiometers. (2) Measurement protocols were not exactly the same.

Results from both Preventiometers were largely consistent. At least acceptable ICCs (> 0.70) were found for all variables except for *blood pressure* measurements, where ICCs between 0.5 and 0.6 occurred. In both Preventiometers, blood pressure measurements were higher compared to their SHIP counterparts whereas the opposite was true for *spirometric measurements*.

Table 5 displays an overview of results from method comparison studies with similar variables. Four studies reported ICCs and/or bias and limits of agreement for *somatometric* variables. The observed mean differences in our study for *body height*, *body weight*, *hip,* and *waist* measurements are not larger in comparison but the limits of agreement for *hip* and *waist* measurements are. The latter indicates the presence of more unsystematic measurement error in the Preventiometer assessment.

**Table 5:** Agreement and validity estimates from similar method comparison studies

Authors	Device(s)	Comparison	Variable	Subgroup	n	Bias	LoA^a^	ICC
Jaeschke et al. 2015 [40]	Vitus Smart XXL	Manual measurement vs. Bodyscanner (see device)	height	Men	27	0.6	1.8	
			waist	Men	27	1.5	5	
			hip	Men	27	2.3	2.6	
			height	Women	32	1.2	2	
			waist	Women	32	4.7	6.6	
			hip	Women	32	3	3.6	
McEneaney & Lennie 2011 [41]		Self- vs. professional measurement / Self-measurement: Written vs. video instructions	waist	Written instructions	29	1.75	6.4	
			hip	Written	29	-0.35	7.2	
			waist	Video	28	0.95	8	
			hip	Video	28	-0.75	6.65	
Dekkers et al. 2008 [42]		Self-reported vs. manual measurement	height		1298	-0.7	3	.99
			weight		1298	1.4	3.8	1
			waist		1298	-1.1	7.9	.96
Ross & Wing 2016 [43]	BodyTrace smart scale, BWB-800	Home scale vs. clinical measurement	weight		58	1.1	1.6	
Jensky-Squires et al. 2008 [44]	BioSpace InBody 320 (BIA1), Omron (BIA2), Bod-eComm (NIA), dual-energy X-ray absorptiometry (DXA)	BIA1 vs. DXA	body fat%	Men	254	-2	6.4	
		BIA2 vs. DXA	body fat%	Men	217	-1	8.4	
		NIA vs. DXA	body fat%	Men	252	-1.8	9.4	
		BIA1 vs. DXA	body fat%	Women	254	-3.6	6.6	
		BIA2 vs. DXA	body fat%	Women	217	-3.3	7.8	
		NIA vs. DXA	body fat%	Women	252	-4.8	10.5	
Williams & Bale 1998 [45]	Harpenden skinfold caliper (SKF), BIA 101 (BIA), Hydrostatic weighing (HYD)	HYD vs. BIA	body fat%	female	115	-1.2	4.9	
		HYD vs. SKF	body fat%	female	115	-1.4	4.4	
		HYD vs. BIA	body fat%	male	117	0.6	4	
		HYD vs. SKF	body fat%	male	117	0.1	3.7	
Cassidy & Jones 2001 [46]		right-left arm, 1st vs. 2nd measurement	Syst. BP		237	4.77	27	
			Diast. BP		237	3.73	20.6	
Christofaro et al. 2009 [47]	Omron MX3 Plus vs. Mercury	ICC from first measurement, measurement were taken simultaneously	Syst. BP		165	2.1	10.1	.80
			Diast. BP		165	0.8	10.3	.71
Agarwal 2016 [48]	Omron HEM 907, Omron HEM 705 CP	Measurement under research vs. clinical conditions	Syst. BP		275	-12.7	33.4	
			Diast. BP		275	-12	22.1	
Vera-Cara et al. 2011 [49]	Omron HEM-705-CP	mean of 3 auscultatory vs. mean of 2 oscillometric measurements	Syst. BP		1084	1.80	11.9	
			Diast. BP		1084	-1.60	10.8	
Smith & Hofmeyr 2019 [50]	Contec CMS50D, Nihon Kohden Life Scope MU-631 RK	Fingertip pulse oximeter vs. conventional bedside monitor	Heart rate		220	-0.43	5.2	
Mitchell et al. 2016 [4]	Polar watch, Android App, iPhone App	Polar vs. android	Heart rate		111	-1.75	7.5	.95
		Polar vs. iphone	Heart rate		111	-1	5.9	.97
Losa-Iglesias et al. 2016 [28]	Radial pulse (RAD), Nonin GO2 (OXI), Heart Rate Plus (APP)	RAD vs. OXI	Heart rate		46	-0.21	2.95	.99
		RAD vs. APP	Heart rate		46	3.12	5.12	.95
		OXI vs. APP	Heart rate		46	3.24	5.21	.94
Liistro et al. 2006 [51]	Vmax 20C, Morgan TLC, Datospir 70, Microloop, Spirobank	Datospir 70	FVC^b^		399	-0.07	0.54	
		Microloop	FVC^b^		399	-0.03	0.44	
		Spirobank	FVC^b^		399	-0.04	0.52	
Gerbase et al. 2013 [52]	SM 2200 (SM), EasyOne handhelds (EO1-EO3)	EO1 vs. SM	FVC^b^		82	-0.13	0.31	
		EO2 vs. SM	FVC^b^		82	-0.02	0.3	
		EO3 vs. SM	FVC^b^		82	-0.07	0.3	
Wiltshire & Kendrick 1994 [53]	Escort spirometer, Model S wedge bellows spirometer, Wright peak flow meter	Escort vs. Wedge	FVC^b^		113	0.03	0.56	
		Escort vs. Wright	PEF		113	0.03	1.7	
Swart et al. 2003 [54]	Spirospec desktop spirometer, Masterlab 4.0 standard spirometer		FVC^b^		45	0.03	0.24	
			PEF		45	-0.41	1.12	
Rebuck et al. 1996 [55]	Welch-Allyn Pneumocheck, P.K. Morgan Sprioflow 12		FVC^b^		75	0.06	0.56	
			PEF		75	0.44	1.9	
Maree et al. 2001 [56]	Diagnosa, Masterlab 4.0		FVC^b^		45	-0.1	0.22	
			PEF		45	-0.03	1.18	
Fonseca et al. 2005 [30]	PiKo-1, Spirotel, Mini-Wright, Vitalograph 2120 (reference)	PiKo-1 vs. reference	PEF		38	0.22	1.48	.90
		Spirotel vs. reference	PEF		38	-0.35	1.53	.95
		Mini-Wright vs. reference	PEF		38	-1.15	2.9	.87

*n* number of participants, *LoA* limit of agreement, *ICC* intraclass correlation coefficient

^a^Because detailed information about limits of agreement often lacks, we report the crude LoA, computed as the twofold standard deviation of the differences, here

^b^We only include the comparisons of spirometers with a turbine as flow sensor (as in our studies) to the standard spirometers

Method comparison studies related to *blood pressure* measurement reported a wide range of agreement indices depending on the compared methods, the context of measurement, and the duration between measurements. Bias and limits of agreement we observed in our study lie at the upper end compared to these studies. The strict criterion proposed by the European Society of Hypertension according to which 95% limits of agreement should not exceed 15 mmHg was not met [57]. The observed differences may be explained by the procedural differences as outlined above, particularly the lack of a systematic resting period prior to the measurements due to the interest of shortening the examination time, and the time-interval between Preventiometer and SHIP measurements.

ICCs for *body fat* seemed relatively low when compared to other measures. A study comparing near-infrared interactance (NIA)—the same method as implemented in the Preventiometer—and dual-energy X-ray absorptiometry (DXA) *body fat* measurement reported absolute bias and limits of agreement that fall into the same range as the present study [44]. However, the same study reported smaller absolute bias values and narrower limits of agreement when comparing bioelectrical impedance analysis (BIA) to DXA. In another study comparing BIA and calipometry to hydrodensitometry, even smaller bias values and narrower limits of agreement are reported [45]. The ICCs reported in a validation study evaluating the agreement between a commercial bioelectric impedance scale and calipometry are much higher than in the present study. Thus, our results are comparable to other studies using NIA, but better results might be achieved by using alternative methods of *body fat* measurement (BIA or calipometry).

Bias for *heart rate* measurement is comparable to other studies, yet, limits of agreement in our study are much larger while ICCs are lower. This might be due to the comparatively large time-interval between the Preventiometer and SHIP measurements and the lack of a resting phase before measurements in the Preventiometer.

Regarding spirometric measurements, estimates of bias and limits of agreement found in Study 2 were at the upper end of the range of what has been found in similar studies. One study also reports ICCs for *peak flow* measurements that are slightly higher than ICCs obtained in our study [24].

## General discussion

Overall, while Preventiometer examinations have adequate reliability according to conventional cut-offs [16], which are in line with results from comparable methods studies (Table 3): Yet, there are some issues to be overcome to increase the comparability of results to the conventional assessment of the studied biomarkers in a cohort study. Measurement agreement was acceptable for most examinations with the exception of *blood pressure.* The consistently higher blood pressure measurements in the Preventiometer may be dealt with by introducing a larger resting period before, and by repeating measurements. In addition, the limits of agreement for most examinations were large compared to other method comparison studies dealing with similar variables. This likely reflects a relevant influence of random measurement error which is also supported by the fact that variance components of methods were consistently smaller than variance components of residuals in the ICC models, respectively. However, one has also to take into account the natural clinical outcome: For example, systolic blood pressure, diastolic blood pressure, and pulse rate can be expected to have lower agreement than body fat or body weight because the underlying physiological magnitudes and processes are more volatile [58]. Thus, the comparatively low ICCs and large limits of agreement for *blood pressure* and *heart rate* may be partly explained by this variability. Another source of disagreement is probably rooted in the methodological and procedural differences described in the discussions of Study 1 and Study 2 (e.g., resting phases, time-intervals). Therefore, a better agreement between *blood pressure* measurements in Preventiometer and SHIP may be expected, if the procedures were harmonized.

In contrast to *blood pressure* and *heart rate,* natural variability may not explain discrepancies with regards to *body fat* measurements. The body fat measurement device in the Preventiometer only measures body fat values up to 45% whereas the Bod Pod (SHIP) does not have this technical measurement limit. Inspecting the Bland–Altman Plots for the comparisons of body fat measurement, this problem becomes visible in form of the points lying on the decreasing line at the right end of the plot. However, we decided to not exclude these data points since this problem may arise in many application contexts with normal populations (which also include people with body fat percentages above 45%) and thus, this technical measurement limit also impairs the validity.

To improve the comparability of the Preventiometer results, we suggest the following steps: (1) *Blood pressure* measurement should follow procedures of available guidelines [59], that is at least two successive measurements shall be obtained and a resting pause of 5 min should be implemented before the first measurement. (2) *Spirometry* should be extended by the inspiratory part of the examination as recommended in relevant guidelines. This has been already implemented in the course of PAKt-MV. (3) The *body fat* measurement device should be replaced by a more valid device. The actual near-infrared interactance body fat device not only has considerable disagreement with the Bod Pod device from SHIP but it also has a technical measurement limit at 45% (see above). While near-infrared interactance is a very time-efficient measurement method to assess body fat, one should keep in mind that it is usually applied to one body point only, while the more valid and traditional skinfold method is applied to multiple body points and an algorithm is used to compute overall body fat [60]. Therefore – technical limitations notwithstanding, multiple body points might be measured with the near-infrared interactance method, thereby combining the time-efficiency of the near-infrared interactance method with the validity of the skinfold method. However, testing the validity using multiple vs. single measuring points with the near-infrared interactance method, Heyward et al. [61] found only a small advantage using multiple measuring points.

### Limitations

Repeated measurements within a single study would have allowed for a variance decomposition and better estimation of the measurement error (a) due to the Preventiometer, (b) due to SHIP, and (c) due to the lack of agreement between Preventiometer and SHIP. However, logistical constraints required that SHIP participants could only be examined once, allowing for no variation of the sequential order of Preventiometer and SHIP examinations in Study 2, and the Preventiometer examinations always took place after the SHIP examinations. We did not cover all potential measurements of the Preventiometer [5, 6] because we focused on measurements comparable to SHIP. Measurement properties are of relevance to provide an informed overview on the usefulness of the Preventiometer for participants and researchers alike. Yet, other aspects beyond the scope of this paper are of relevance as well. The positive user experience [7, 8] has been commented upon. We were also able to perform assessments right at the work place of participants, resulting in little to no travel time for them. Effects on response would need to be dealt with in a separate study. Another aspect is a formal comparison of staffing requirements. When using a bus, there must be a driver with an appropriate license. Overall, compared to stationary examinations, there may be little options to save personnel. On the other hand a very important issue is resolved. All data is collected electronically and stored in a single database. Therefore, background IT-infrastructure is provided, which is important from a provider perspective. In addition, a larger follow-up study is recommended, once the issues raised here have been resolved.

### Conclusion

The initial motivation of these studies was to evaluate the Preventiometer for the use in a preventive health care project (PAKt-MV). As previously stated, reliability is a prerequisite for the detection of change within subjects over time. In our current evaluation, we found the Preventiometer’s measurements sufficient in this regard. However, measurement agreement was insufficient for some measurements. While issues like the body fat measurements can be easily remedied by replacing the measurement device, the deviant blood pressure and pulse measures are an indication for a procedural issue. One of the reasons to use the Preventiometer is to save examination time, which benefits the examiners and the participants. To forgo the recommended resting periods for measuring blood pressure and pulse rate can be seen as a trade-off exchanging validity for time. Our findings suggest that insufficient resting periods have a strong biasing impact making a rather conservative point of trade-off to be preferable. Overall, methodological and technological improvements should be realized before using the Preventiometer in population-based research.

## Supplementary Information

**Additional file 1.**

## Data Availability

Data of the SHIP studies and associated projects are available upon reasonable request from the Transferstelle für Daten- und Biomaterialienmanagement [Office for transfer of data and bio materials] and can be applied for under: https://transfer.ship-med.uni-greifswald.de/FAIRequest/data-use-intro

## Abbreviations

BIA: Bioelectrical impedance analysis
DXA: Dual-energy X-ray absorptiometry
FVC: Vital capacity
HYD: Hydrostatic weighing
ICC: Interclass correlations coefficient
IMS: Ipex management software
LoA: Limit of agreement
NIA: Near-infrared interactance
PAKt-MV: Prävention für Arbeitnehmer zur Reduktion von Krankheitstagen durch Motivation und Verhaltensänderung [preventive healthcare for workers with the aim to reduce absenteeism by motivation and behavior]
PEF: Peak flow
PROMs: patient-reported outcome measures
RAD: Radial pulse
SEM: Standard error of measurement
SHIP: Study of Health in Pomerania
SKF: skinfold caliper
SMD: standardized mean difference
VC: Variance components
