# Supplementary material for: The Preventiometer - reliability of a cardiovascular multi-device measurement platform and its measurement agreement with a cohort study
Source: BMC Med Res Methodol. 2023 Apr 24;23:103. doi: 10.1186/s12874-023-01911-x (PMC10127382; doi:10.1186/s12874-023-01911-x)
Supplement: Supplementary file 1 — Additional file 1. [file 12874_2023_1911_MOESM1_ESM.docx]

**Supplementary Material for**

***The Preventiometer - Reliability of a cardiovascular multi-device measurement platform and its measurement agreement with a cohort study***

In Study 2 one data pair for the weight in kg for stationary Preventiometer was excluded, while for the mobile Preventiometer 2 data pairs for weight in kg and 2 data pairs for height in cm were excluded. The corresponding Blend-Altman plots are compared to provide a visualization of differences between data with and without this correction (see Figure a).

|  | **with correction (as in paper)** | **without correction** |
| --- | --- | --- |
| **weight in kg for stationary Preventiometer** | 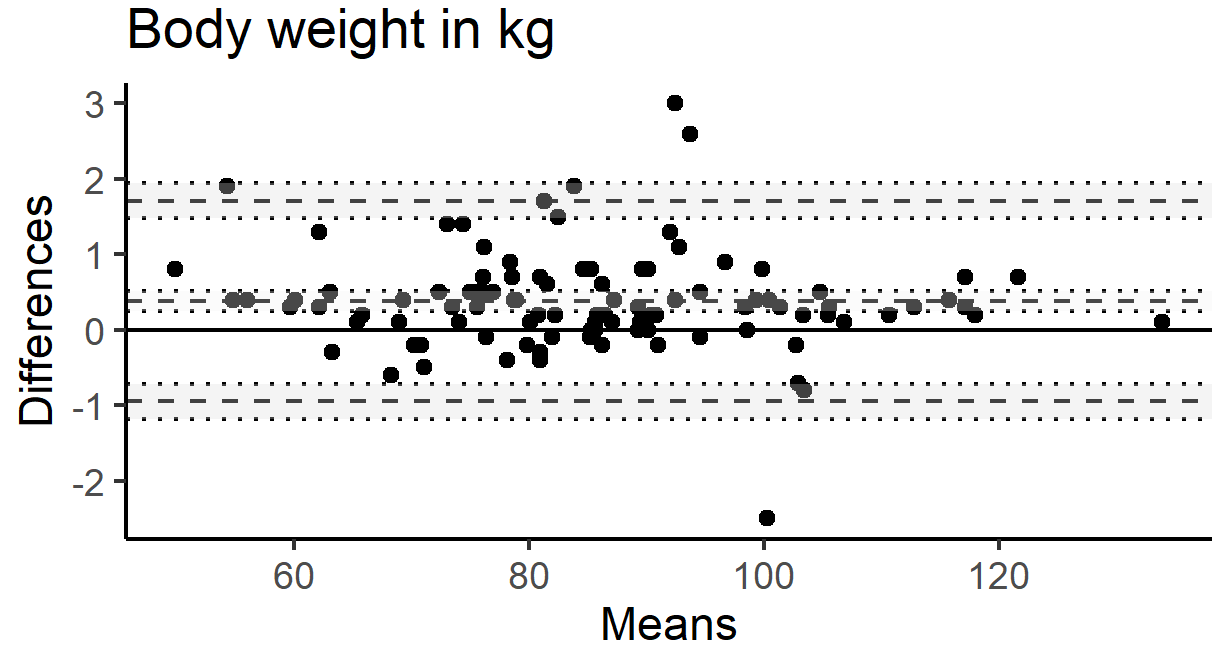 | 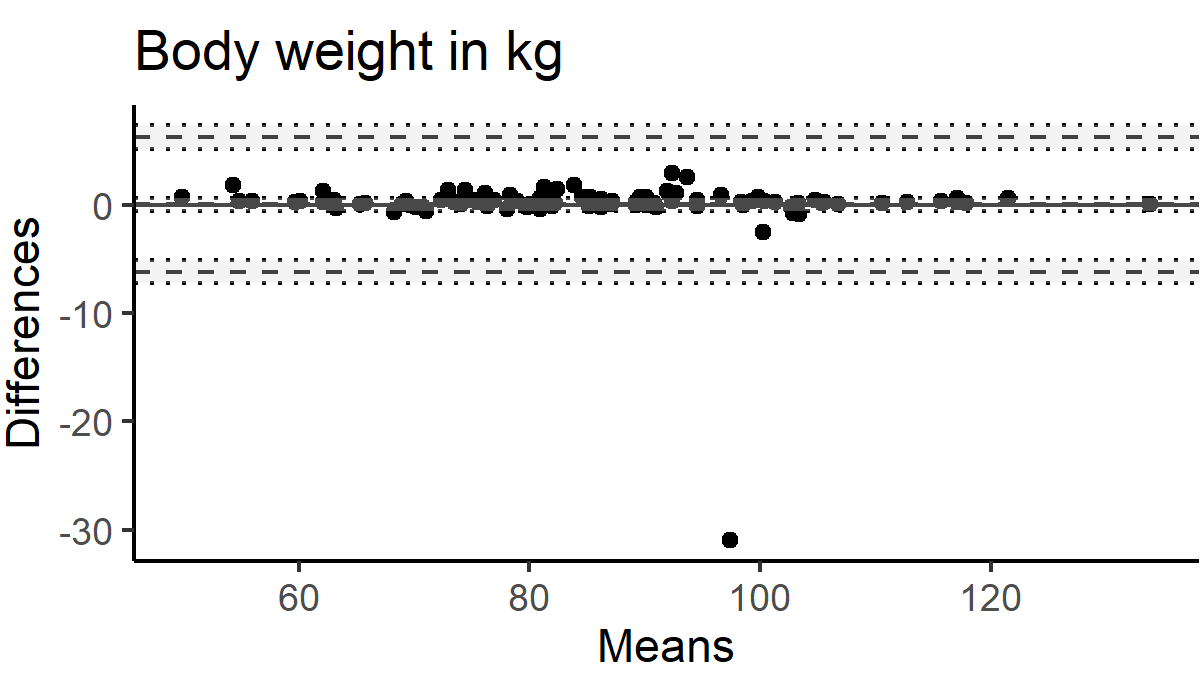 |
| **weight in kg for mobile Preventiometer** | 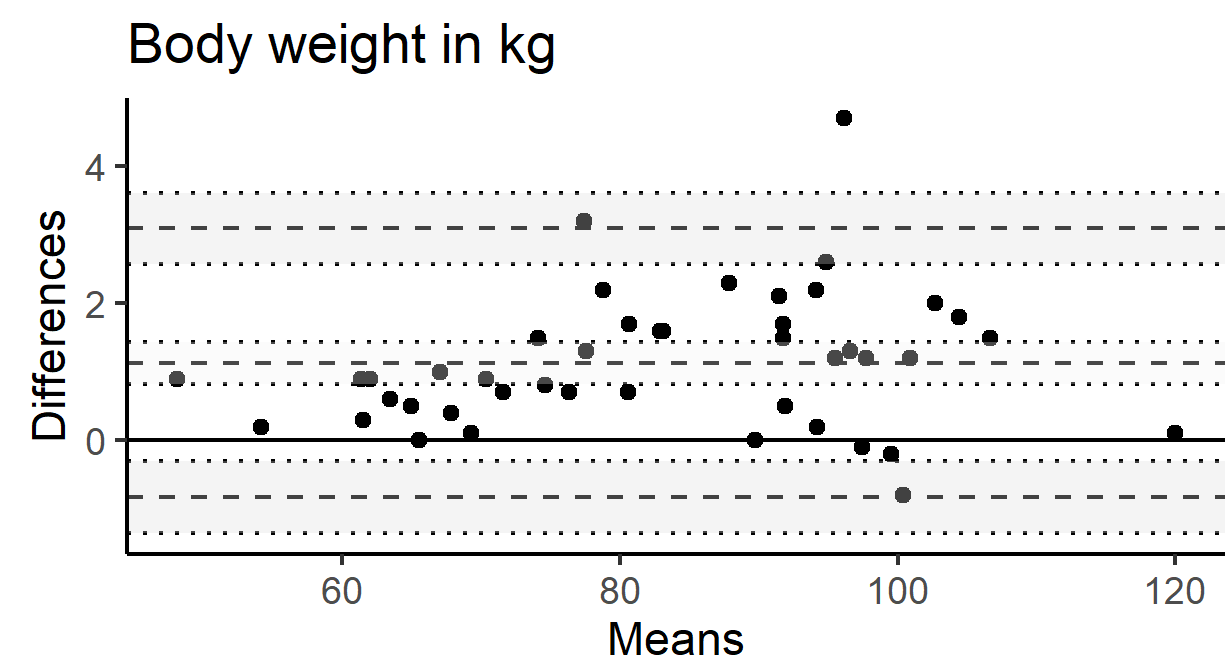 | 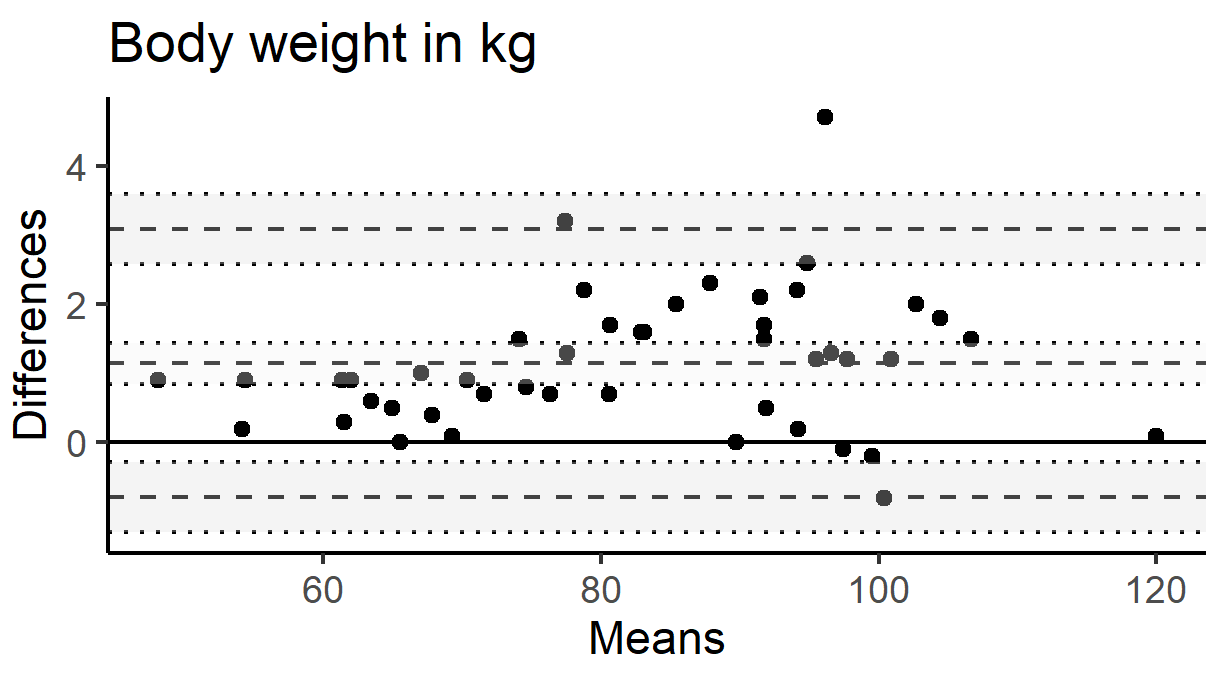 |
| **height in cm for stationary Preventiometer** | 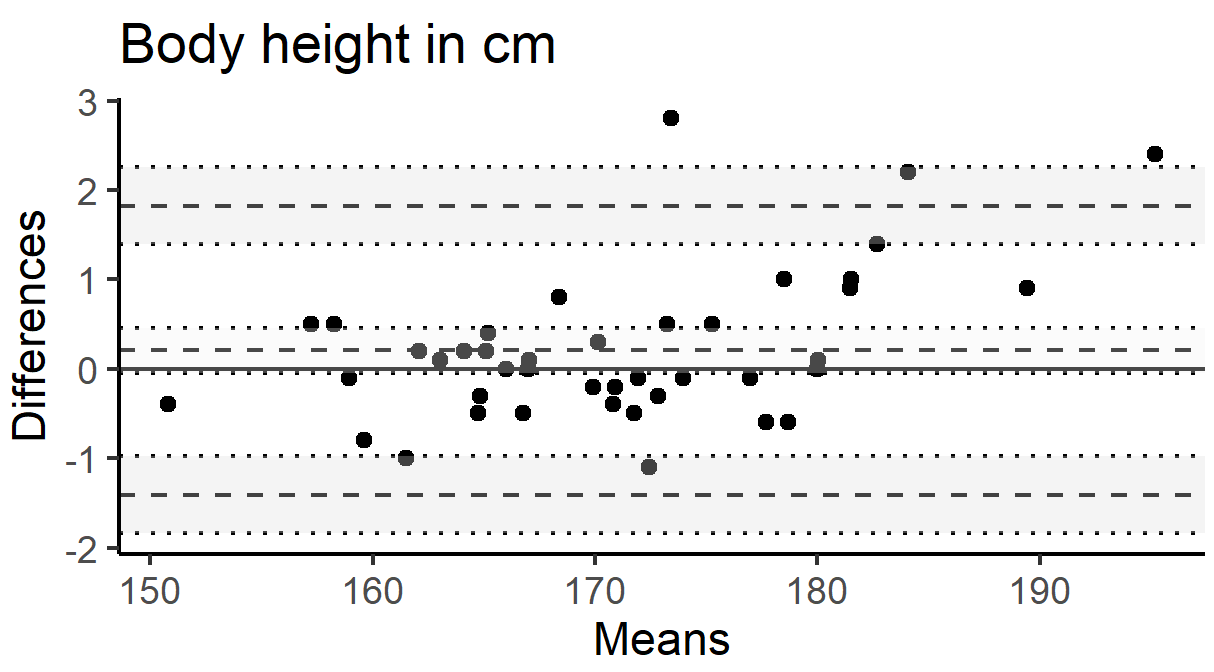 | 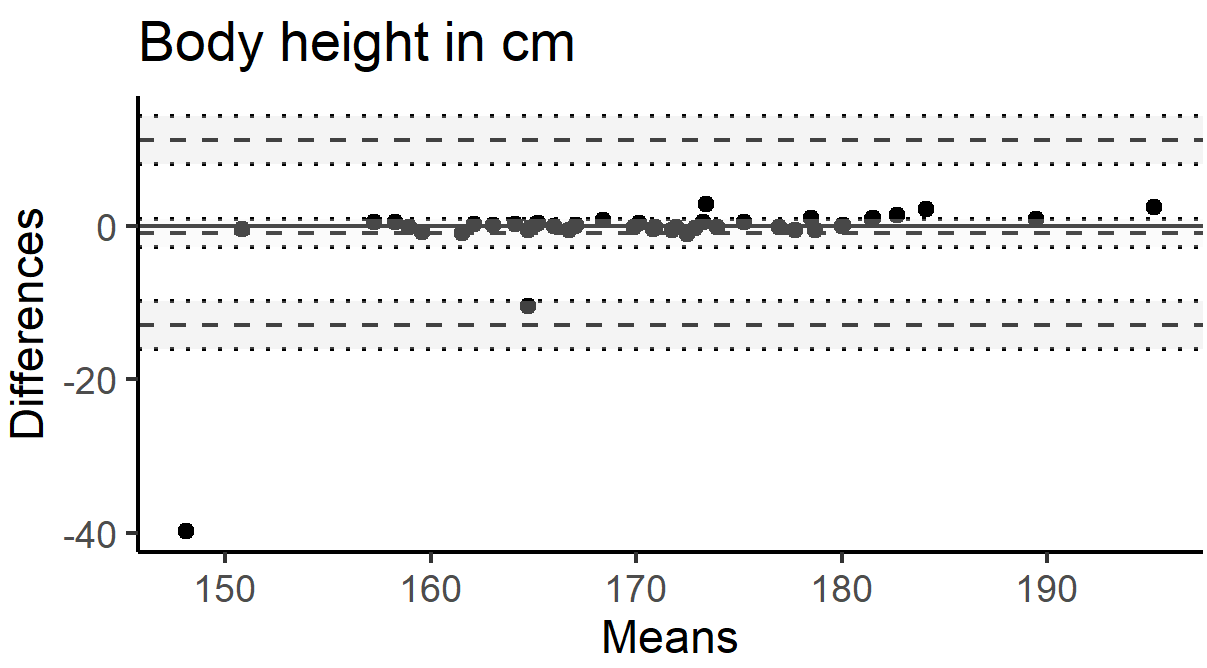 |

*Figure A.* Blend-Altman plots for the comparison of the effects of data exclusion.
